# Cerebellum and hippocampus abnormalities in patients with insomnia comorbid depression: a study on cerebral blood perfusion and functional connectivity

**DOI:** 10.3389/fnins.2023.1202514

**Published:** 2023-06-16

**Authors:** Minghe Xu, Qian Wang, Bo Li, Shaowen Qian, Shuang Wang, Yu Wang, Chunlian Chen, Zhe Liu, Yuqing Ji, Kai Liu, Kuolin Xin, Yujun Niu

**Affiliations:** ^1^Postgraduate Training Base of the 960th Hospital of People's Liberation Army Joint Logistic Support Force, Jinzhou Medical University, Jinan, China; ^2^Department of Radiology, Qingdao Hospital of Traditional Chinese Medicine (Qingdao Hiser hospital), Qingdao, China; ^3^Department of Radiology, The 960th Hospital of People's Liberation Army Joint Logistic Support Force, Jinan, China; ^4^Sleep Clinic, The 960th Hospital of People's Liberation Army Joint Logistic Support Force, Jinan, China; ^5^Department of Radiology, First Affiliated Hospital of Jinzhou Medical University, Jinzhou, China

**Keywords:** insomnia, depression, cerebral blood flow, functional connectivity, arterial spin labeling, resting-state functional magnetic resonance imaging

## Abstract

Chronic insomnia disorder and major depressive disorder are highly-occurred mental diseases with extensive social harm. The comorbidity of these two diseases is commonly seen in clinical practice, but the mechanism remains unclear. To observe the characteristics of cerebral blood perfusion and functional connectivity in patients, so as to explore the potential pathogenesis and biological imaging markers, thereby improving the understanding of their comorbidity mechanism. 44 patients with chronic insomnia disorder comorbid major depressive disorder and 43 healthy controls were recruited in this study. The severity of insomnia and depression were assessed by questionnaire. The cerebral blood perfusion and functional connectivity values of participants were obtained to, analyze their correlation with questionnaire scores. The cerebral blood flow in cerebellum, vermis, right hippocampus, left parahippocampal gyrus of patients were reduced, which was negatively related to the severity of insomnia or depression. The connectivities of left cerebellum-right putamen and right hippocampus-left inferior frontal gyrus were increased, showing positive correlations with the severity of insomnia and depression. Decreased connectivities of left cerebellum-left fusiform gyrus, left cerebellum-left occipital lobe, right hippocampus-right paracentral lobule, right hippocampus-right precentral gyrus were partially associated with insomnia or depression. The connectivity of right hippocampus-left inferior frontal gyrus may mediate between insomnia and depression. Insomnia and depression can cause changes in cerebral blood flow and brain function. Changes in the cerebellar and hippocampal regions are the result of insomnia and depression. They reflect abnormalities in sleep and emotion regulation. That may be involved in the pathogenesis of comorbidity.

## 1. Introduction

Insomnia is a mental disorder with a high morbidity. Approximately 13.9% adults are suffering from insomnia (Morin et al., 2020). Insomnia with a course of more than 3 months is known as chronic insomnia disorder (CID). More patients with CID will have depression after long-term insomnia, and then develop major depressive disorder (MDD). Therefore, CID and MDD may be comorbidities, but their mechanisms remain unknown. Generally, insomnia is caused by the dysfunction of sleep regulation of the central nervous system, which is manifested as an abnormal hyperawakening state of the brain. The regulation of sleep and wakefulness is often the result of synergistic effect of multiple brain regions, while the occurrence of insomnia is usually closely related to their abnormal function. Long-term insomnia often results in the possibilities of depression, anxiety and other mental disorders in insomnia patients (Lancel et al., 2021). Currently, a comorbidity between insomnia and depression may be attributed to their similar pathological mechanisms and neural pathways. Research on the brain of CID patients have found abnormal changes in brain areas related to emotional regulation. Studies in MDD patients have shown abnormal changes in brain areas related to sleep regulation.

Previous studies have found abnormal regional cerebral blood flow (CBF) in patients with insomnia and depression. In sleep deprivation experiments (Rao, 2012; Zhou et al., 2019), researchers found significant reductions of CBF in the hippocampus, prefrontal, and amygdala, which gradually disappeared when sleep was restored. In studies on patients with insomnia and depression (Park et al., 2021), abnormal changes of CBF were observed in insula, cerebellum and orbitofrontal cortex, and CBF was found correlated with sleep quality, depression severity, and anxiety severity. Functional connectivity (FC) analysis based on blood oxygenation-level dependent imaging showed different research value, including the ability to reflect the activity of neurons in regional brain, verify FC changes in brain regions with abnormal CBF, and preferably reveal the pathogenesis of CID and MDD. Many studies have found that FC changes exist in some brain regions of patients with insomnia or depression. From the perspective of brain network theory, researchers found that the default mode network, negative emotional network and reward network abnormalities are closely related to insomnia and depression (Leistedt and Linkowski, 2013; Marques et al., 2018).

Our research focuses on CID comorbidity MDD, which is a mental disorder with numerous patients. In previous studies, insomnia and depression were generally regarded as two independent diseases while disregarding their simultaneous occurrence. We strive to investigate the potential pathogenesis and comorbidity mechanism of CID and MDD and find effective biomarkers of images, so as to shed new light on the next research.

## 2. Materials and methods

### 2.1. Participants

44 patients with CID comorbid MDD were recruited from the Sleep Clinic of the 960th Hospital of the People’s Liberation Army Joint Logistic Support Force (male = 22, female = 22) (34 ± 7 years, ranging from 21 to 50 years). All patients were diagnosed by two experienced doctors mainly according to the Diagnostic and Statistical Manual of Mental Disorders Fifth Edition. They were assessed for mental status and health to meet the experimental requirements. All patients were confirmed to have initially experienced insomnia and then developed depressive mood. The inclusion criteria are as follows: (a) The diagnosis was in line with the Diagnostic and Statistical Manual of Mental Disorders Fifth Edition criteria for insomnia disorder and MDD; (b) Duration of insomnia at least 3 times a week, lasting for 3 months or more; (c) Pittsburgh Sleep Quality Index (PSQI) scores >7, and Insomnia Severity Index (ISI) scores >15; (d) Back Depression Inventory-21 items (BDI) scores >20, Hamilton Depression Scale-24 items (HAMD) scores >21; (e) Age 18–55 years; (f) Han nationality and right-handedness. The exclusion criteria were as follows: (a) Other sleep disorders; (b) Any other neurological, psychiatric, or somatic disorders; (c) History of any hypnotic and antidepressant medication 2 weeks before neuropsychological test and MRI scan; (d) History of substantial head trauma and routine MRI showed abnormal brain structure; (e) Drug, alcohol or cigarette use 24 h before the scanning; (f) Abuse of alcohol or drugs; (g) Female who were regnant, lactating, or menstruating; (h) Contraindications to MRI. (i) Neurological and motor function abnormalities.

We also recruited 43 healthy controls (HC) with similar age, gender, and education years distribution from local communities via advertisements (male = 22, female = 21) (34 ± 8 years, ranging from 19 to 48 years). Participants were screened through diagnostic interviews, namely, Structured Clinical Interview for DSM-5 Nonpatient Edition, so as to exclude any current or past history of psychiatric disorders. In addition, all healthy controls reported favorable sleep quality and mental status. No brain lesions were found by T2 MRI. In order to assess the sleep quality and anxiety of patients, participants completed PSQI, ISI, BDI-21, and HAMD-24 questionnaires.

The present study was approved by the Research Ethics Committee of the 960th Hospital of the People’s Liberation Army Joint Logistic Support Force and accorded with the Declaration of Helsinki. Participants volunteered to participate in the study and signed a written informed consent after receiving a complete written and verbal explanations regarding the study.

### 2.2. MRI data acquisition

MRI scanning was performed on a GE MR750 3.0 T scanner (General Electric, Milwaukee, Wisconsin) featuring an 8-channel phased-array head coil. Participants lay supine with their heads secured using a belt and foam pads to minimize head movements. During the resting-state scan, participants were instructed to close their eyes, think about nothing or falling asleep, which was confirmed after the scan was completed.

Rs-fMRI data were acquired using gradient echo-planar imaging. Each scan consisted of 260 gradient echo-planar volumes with the parameters of: repetition time (TR) = 2000 ms, echo time (TE) = 30 ms, flip angle (FA) = 90°, matrix = 64 × 64, field of view (FOV) = 24 × 24 cm^2^, voxel size = 3 × 3 × 3 mm^3^, thickness/gap = 4/0 mm, lasting 8 min 40 s. Three-dimensional brain volume imaging sequence covering the whole brain was used for structural data acquisition with the parameters as follows: 132 slices; TR = 8.2 ms, TE = 3.2 ms, slice thickness = 1.0 mm, FOV = 24 × 24 cm^2^, resolution =256 × 256, FA = 12°. The CBF images were acquired with 3D pseudocontinuous ASL sequence, with the following parameters: TR = 4,632 ms, TE = 10.5 ms, slice thickness = 4 mm, matrix =128 × 128, FOV = 24 × 24 cm^2^, post labeling delay = 1,525 ms, and each spiral arm included 512 sampling points in k-space and a total of 8 arms were acquired. Routine MRI confirmed no brain structural abnormalities. Two experienced radiologists confirmed that all participants were free of structural brain abnormalities and significant head motions on routine MRI.

### 2.3. Measurement of insomnia and depression severity

The insomnia severity of the participants was assessed using PSQI and ISI scores. Depression severity was assessed by HAMD and BDI scores. The cognitive function was assessed using the Montreal Cognitive Assessment Scale (MoCA).

### 2.4. ASL MRI data processing

ASL MRI images of each participant were collected using an automated image postprocessing tool (Functool, version 12.2.01), which was embedded in the GE MR750 scanner system. After subtracting the label image from the control images, we obtained the absolute quantification map for each participant. Subsequently, the CBF and anatomical images were processed with Statistical parametric mapping (SPM12; http://www.fi.ion.ucl.ac.uk/spm/) and Rest1.8 toolbox. (1) The individual CBF images were realigned to correct head motio, (2) The individual CBF images were registered to the corresponding anatomical image, and the registration parameters were used to transform the individual CBF maps into the space of the anatomical image, (3) The anatomical images were then normalized to the Montreal Neurological Institute (MNI) template, (4)The individual CBF images were normalized into a 3 mm × 3 mm × 3 mm in MNI space, (5) The individual CBF images were spatially smoothed with a 4 mm × 4 mm × 4 mm full width at half maximum Gaussian kernel, and (6) Entering into the whole brain CBF analysis in a voxel-wise manner.

Two-sample *t*-tests and whole-brain voxel-wise analyses were used to compare the smoothed CBF maps between the two groups. Age, gender, years of education and mean global CBF of gray matter were included as covariates. Gaussian Random Field (GRF) correction was used for multiple comparisons with corrected threshold of voxel value of *p* of <0.005 and cluster value of *p* of <0.05. Finally, CBF values of the brain areas with significant changes were extracted.

### 2.5. FC data processing

Statistical parametric mapping (SPM12; http://www.fi.ion.ucl.ac.uk/spm/) and Rest1.8 toolbox were used to perform the data preprocessing and statistical analysis. The preprocessing steps of the functional image are as follows: (1) Convert DICOM files into NIFITI images, (2) Discard the first 10 images, (3) Slice remaining images for time correction first, then realign to the first image and register with the average of these images, (4) Rigid-body head motion correction (2 mm displacement and 2 degrees rotation), (5) Segment individual T1 structural images (including white matter, gray matter, and cerebrospinal fluid), (6) Coregister each structural image with the functional image, and then reconstruct it into 3 × 3 × 3 mm^3^ resolution voxels, (7) Spatial normalization of the Montreal Neurological Institute (MNI), (8) Spatial smoothing (4 mm full width at half maximum), (9) Remove the linear trend and perform 0.01–0.1 Hz band-pass filtering, (10) Nuisance covariates (head motion parameters, global mean signal, white matter signal and cerebrospinal fluid signal) were regressed from the data, (11) Define regions with significant group differences that automatically displayed in CBF maps as regions of interest (ROIs) and seed regions, (12) Calculate the connectivity between the seed region and each voxel of the whole brain and, draw correlation maps, and (13) Convert correlation maps into z-values maps using fisher’s r-to-z transformation.

A two-sample t-test was utilized to investigate the FC differences between groups. Age, gender, and years of education were included as covariates. GRF correction was applied to perform multiple comparison correction. The significance threshold was set as voxel level of *p* < 0.001, and cluster level of *p* < 0.05. FC values of significantly changed brain areas were extracted.

### 2.6. Statistical analysis

SPSS 20.0 statistical package (Chicago,IL, United States) was used for statistical analysis. Data were inspected for outliers, bias, and homogeneity of variance to ensure the appropriateness of parametric statistical tests. An independent two-sample t-test was performed to assess the differences between demographic and clinical data, including age, education years, PSQI scores, ISI scores, HAMD scores, and BDI scores. Chi-square test was performed to assess differences in gender. *p* < 0.05 was considered statistically significant.

Pearson’s correlation analysis was performed to examine the relationships between clinical variables (including PSQI, ISI, HAMD, BDI scores) and CBF or FC. Age, gender, and years of education were included as covariates. Then, the relationship between different clinical variables also was examined. The significance was set as *p* < 0.05.

Mediation analysis was performed using PROCESS toolbox in SPSS 20.0. The significance of the mediated effect was assessed using 5,000 bias-corrected bootstrapping. A 95% confidence interval without zero was considered significant. Model 4 of the PROCESS toolbox was used to conduct mediation analysis on insomnia severity, anxiety severity and CBF or FC values.

## 3. Result

### 3.1. Demographic and clinical comparisons

Two patients and one healthy participant were excluded due to head movement during image acquisition. A total of 42 patients and 42 healthy controls were analyzed. There were no significant differences in gender, age, education years or MoCA scorebetween the two groups. ISI, PSQI, HAMD and BDI scores of the patient group were higher than those of the healthy control group (Table 1).

**Table 1:** Demographic and clinical data comparisons.

Characteristics	CID comorbid MDD (*n* = 42)	HC (*n* = 42)	*t*/*χ*^2^	*p* Value
Age (years old)	33 ± 7	33 ± 8	0.00^a^	1.00
Gender (M/F)	21 / 21	21 / 21	0.00^b^	1.00
Education (years)	14 ± 2	14 ± 2	0.40^a^	0.70
MoCA scores	30 ± 0	30 ± 0	0.67^a^	0.51
ISI scores	20 ± 2	1 ± 1	57.96^a^	0.00^*^
PSQI scores	15 ± 2	2 ± 1	46.70^a^	0.00^*^
HAMD scores	31 ± 5	1 ± 1	41.40^a^	0.00^*^
BDI scores	32 ± 4	1 ± 1	54.96^a^	0.00^*^

Values are reported as mean and standard deviation, except for gender. CID, chronic insomnia disorder; MDD, major depressive disorder; HC, healthy control; M, male; F, female; ISI, Insomnia Severity Index Scale; PSQI, Pittsburgh Sleep Quality Index; HAMD, Hamilton Depression Scale; BDI, Back Depression Inventory; MoCA, Montreal Cognitive Assessment Scale. a two-sample t-test, b *χ* 2 -test, *p < 0.05

### 3.2. Correlation analysis of insomnia and depression

**Figure 1:**
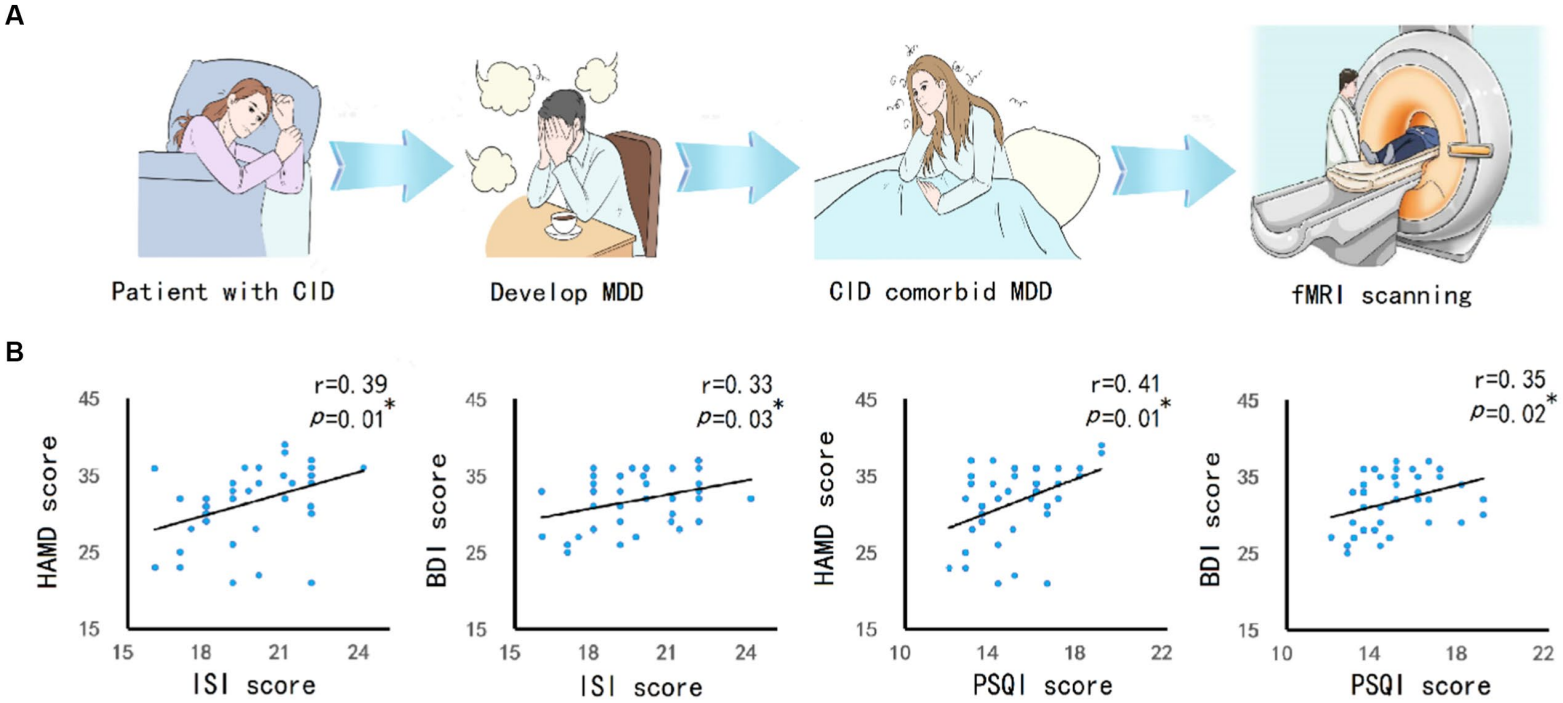
The relationship between CID and MDD. **(A)** The research concept and experimental process. CID patients experienced long-term insomnia, and then developed into MDD. MRI scanning was performed on patients with CID comorbid MDD. **(B)** Pearson’s correlation analysis between insomnia severity (ISI and PSQI score) and depression severity (HAMD and BDI score) in patients showed a positive correlation. The significance was set at *p* value <0.05. * indicates *p* < 0.05. ** indicates *p* < 0.01.

CID may induce MDD, and eventually lead to CID comorbid MDD. The fMRI can be used to detect abnormal changes in the brain (Figure 1A). Correlation analysis (Figure 1B) showed that, the ISI score of patients was significantly positively correlated with HAMD score(*r* = 0.39, *p* = 0.01) and BDI score(*r* = 0.33, *p* = 0.03). The PSQI scores was significantly positively correlated with HAMD score(*r* = 0.41, *p* = 0.01) and BDI score(*r* = 0.35, *p* = 0.02).

### 3.3. Differences In CBF values between groups

**Figure 2:**
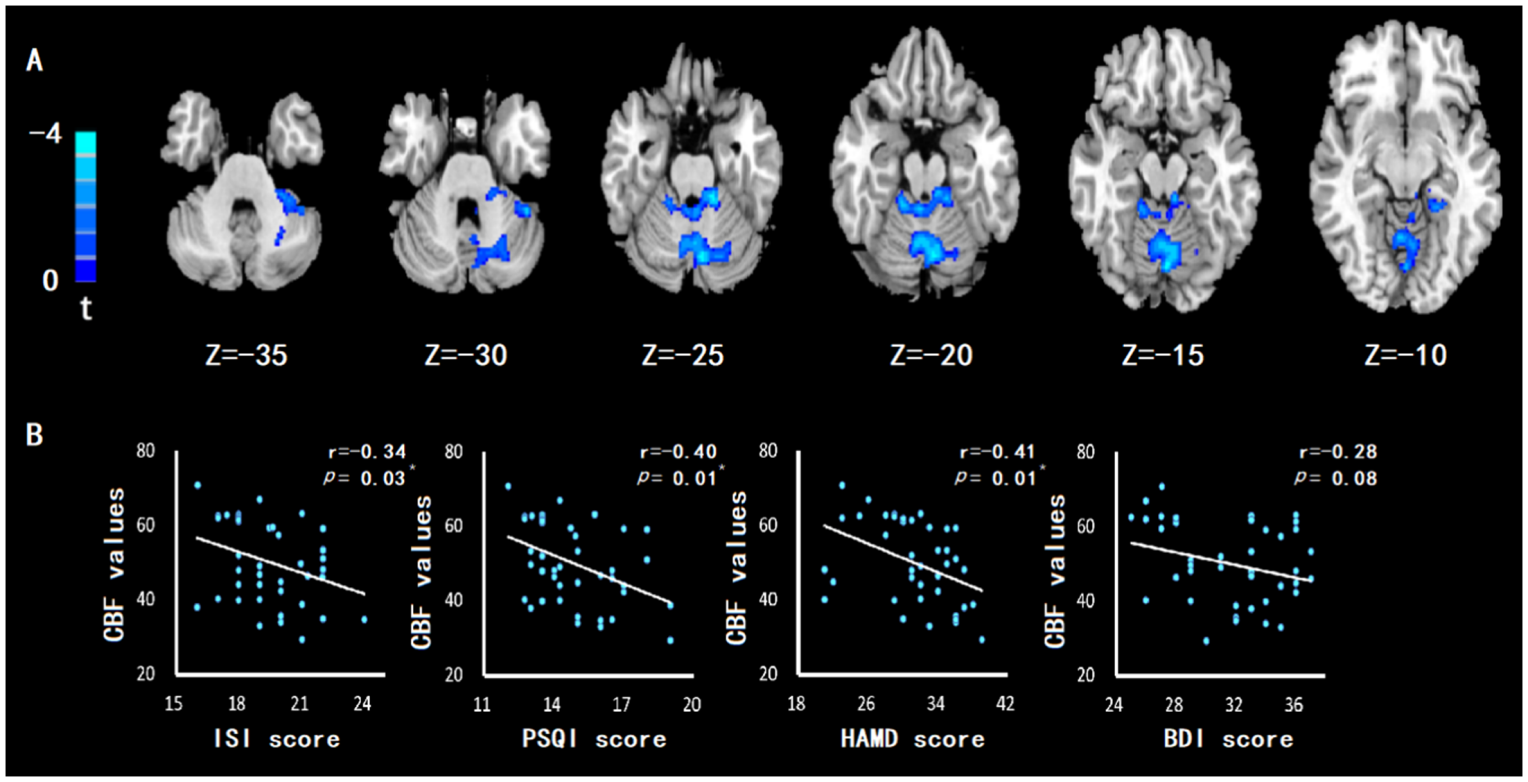
The CBF of cerebellum in patients with CID comorbid MDD was reduced, which was related to insomnia and depression. **(A)** Compared with HC group, patients with CID comorbid MDD showed a wide CBF reduction area, containing part of the left cerebellum, right cerebellum, cerebellum anterior lobe and cerebellum posterior lobe. GRF correction with corrected threshold of voxel *value of p* of <0.005 and cluster *value of p* of <0.05. Significance was set at *value of p* = 0.01. **(B)** The abnormal cerebellar CBF value was negatively correlated with the severity of insomnia (ISI and PSQI score) and depression (HAMD and BDI score). Significance was set at *p* value <0.05. * indicates *p* < 0.05. ** indicates *p* < 0.01.

**Figure 3:**
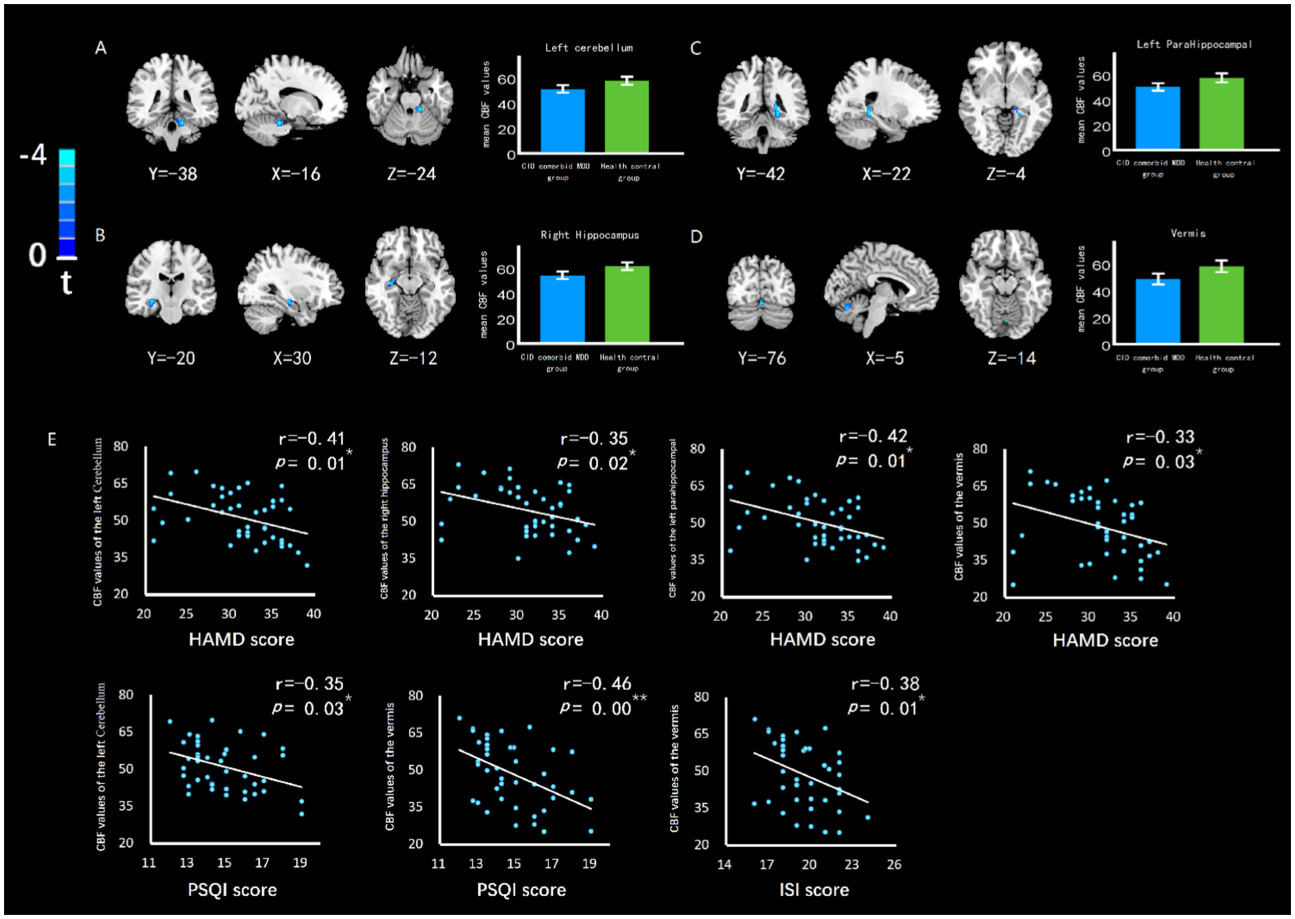
The difference of CBF in brain regions between groups, and the relationship between CBF value and insomnia or depression. The CBF of **(A)** left cerebellum, **(B)** right hippocampus, **(C)** left parahippocampal gyrus, and **(D)** left parahippocampal gyrus of CID comorbid MDD group were reduced significantly. Bar chart showed the difference of CBF value between groups. Errors bar represented the standard error. **(E)** HAMD score was negatively related to CBF of left cerebellum, right hippocampus, left parahippocampal gyrus, vermis. PSQI score was negatively related to CBF of left cerebellum, vermis. ISI score was negatively related to CBF of vermis. * indicates *p* < 0.05. ** indicates *p* < 0.01.

The cerebellum of CID and MDD patients showed wider CBF reduction areas than those of HC group (Figure 2A), which was related to the severity of insomnia and depression (Figure 2B). In addition, the left cerebellum, vermis, right hippocampus, left parahippocampal gyrus also showed significant CBF reduction (Figures 3A-D; Table 2), which was related to the severity of insomnia and depression (Figure 3E).

**Table 2:** Clusters of brain regions showing group differences in cerebral blood flow.

Brain regions	Side	Cluster size (Voxels)	MNI coordinates			*T* value	*p* value
			*X*	*Y*	*Z*		
Cerebellum	L	50	−16	−38	−24	−3.57	0.00^**^
Hippocampus	R	63	30	−20	−12	−3.45	0.00^**^
ParaHippocampal gyrus	L	35	−22	−42	−4	−3.41	0.00^**^
Vermis	L	62	−5	−76	−14	−3.61	0.00^**^

MNI, Montreal Neurological Institute; L, Left; R, Right. *p < 0.05, **p < 0.01.

### 3.4. Differences in FC values between groups

**Figure 4:**
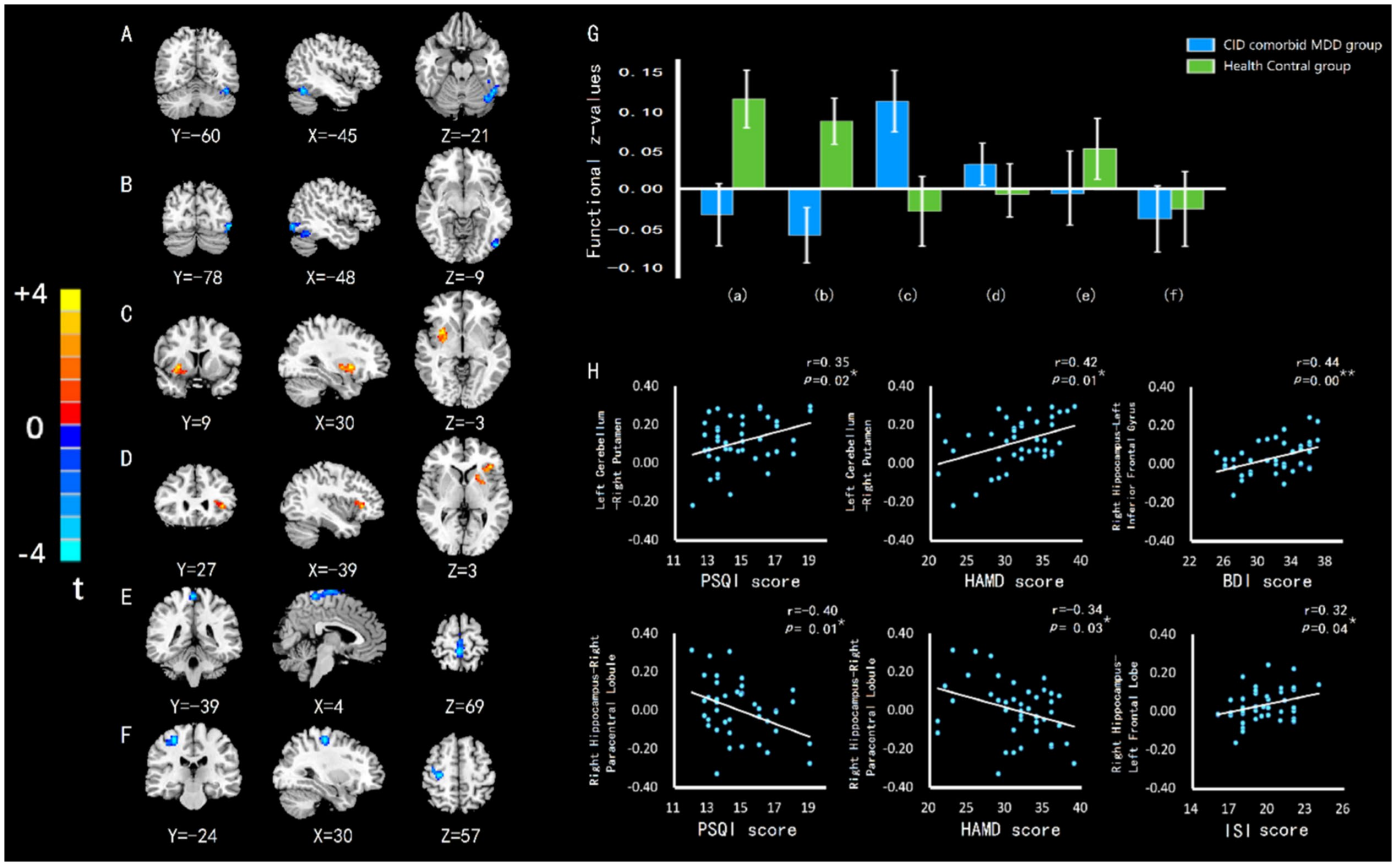
The difference of FC in brain regions between groups, and the relationship between FC value and insomnia or depression. **(A)** FC of left cerebellumleft fusiform gyrus. **(B)** FC of left cerebellum-left occipital lobe. **(C)** FC of left cerebellum-right putamen. **(D)** FC of right hippocampus-left inferior frontal gyrus. **(E)** FC of right hippocampus-right paracentral lobule. **(F)** FC of right hippocampus-right precentral gyrus. **(G)** The differences of FC z-value between groups. Errors bar showed standard error. Labels **(A**-**F)** correspond to the regions with FC difference. **(H)** HAMD or BDI score was positively related to left cerebellum-right putamen connectivity and right hippocampus-left inferior frontal gyrus connectivity in CID comorbid MDD group. HAMD score was negatively related to right hippocampus-right paracentral lobule connectivity. ISI score was positively related to right hippocampus-left inferior frontal gyrus connectivity. PSQI score was positively related to left cerebellum-right putamen connectivity and negatively related to right hippocampus-right paracentral lobule connectivity. *p < 0.05. **p < 0.01.

The left cerebellum and right hippocampus were defined as ROIs. Compared with HC group, patients with CID and MDD exhibited increased connectivity between left cerebellum and right putamen, and increased connectivity between right hippocampus and left inferior frontal gyrus (Brodman 47). Decreased connectivity was found between left cerebellum and left fusiform gyrus, between left cerebellum and left occipital lobe, between right hippocampus and right paracentral lobule, and between right hippocampus and right precentral gyrus (Table 3; Figure 4). Some connectivity differences were related to the severity of insomnia and depression (Figure 4H).

**Table 3:** Clusters of brain regions showing group differences in functional connectivity.

ROIs	Brain regions	Cluster size (Voxels)	MNI coordinates			*T* value	*p* value
			*X*	*Y*	*Z*		
Left Cerebellum	Left fusiform gyrus	28	−45	−60	−21	−4.17	0.01^**^
	Left occipital lobe	29	−48	−78	−9	−5.01	0.01^**^
	Right putamen	60	30	9	−3	3.80	0.01^**^
Right Hippocampus	Left inferior frontal gyrus	20	−39	27	3	3.96	0.00^**^
	Right paracentral lobule	36	0	−39	69	−5.77	0.00^**^
	Right precentral gyrus	62	30	−24	57	−4.50	0.00^**^

ROIs, regions of interest; MNI, Montreal Neurological Institute; L, Left; R, Right. *p < 0.05, **p < 0.01.

### 3.5. Correlation analysis with the clinical characteristics

After correlation analysis of patients, abnormal CBF of left cerebellum, vermis, right hippocampus, left parahippocampal gyrus and wide areas of cerebellum were significantly negatively correlated with the severity of insomnia or depression. The connectivities of left cerebellum-right putamen and right hippocampus-left inferior frontal gyrus were significantly positively correlated with the severity of insomnia and depression. The connectivity of right hippocampus-right paracentral lobule was significantly negatively correlated with the severity of insomnia and depression (Table 4).

**Table 4:** Correlation between CBF/FC values and clinical characteristics.

CBF/FC values	Clinical characteristics	r value	*p* value
Cerebellum (CBF)	ISI	−0.34	0.03^*^
	PSQI	−0.40	0.01^**^
	HAMD	−0.42	0.01^**^
	BDI	−0.28	0.08
Left Cerebellum (CBF)	PSQI	−0.35	0.03^*^
	HAMD	−0.41	0.01^**^
Vermis (CBF)	ISI	−0.38	0.01^*^
	PSQI	−0.46	0.00^**^
	HAMD	−0.33	0.03^*^
Right Hippocampus (CBF)	HAMD	−0.35	0.02^*^
Left ParaHippocampal gyrus (CBF)	HAMD	−0.42	0.01^**^
Left Cerebellum- Right Putamen (FC)	PSQI	0.35	0.02^*^
	HAMD	0.42	0.01^**^
Right Hippocampus- Right Paracentral Lobule (FC)	PSQI	−0.40	0.01^**^
	HAMD	−0.34	0.02^*^
Right Hippocampus- Left Inferior Frontal Gyrus (FC)	ISI	0.32	0.03^*^
	BDI	0.44	0.00^**^

CBF, cerebral blood flow; FC, functional connectivity; ISI, Insomnia Severity Index Scale; PSQI, Pittsburgh Sleep Quality Index; HAMD, Hamilton Depression Scale; BDI, Back Depression Inventory. *p < 0.05, **p < 0.01.

### 3.6. Mediation analysis

**Figure 5:**
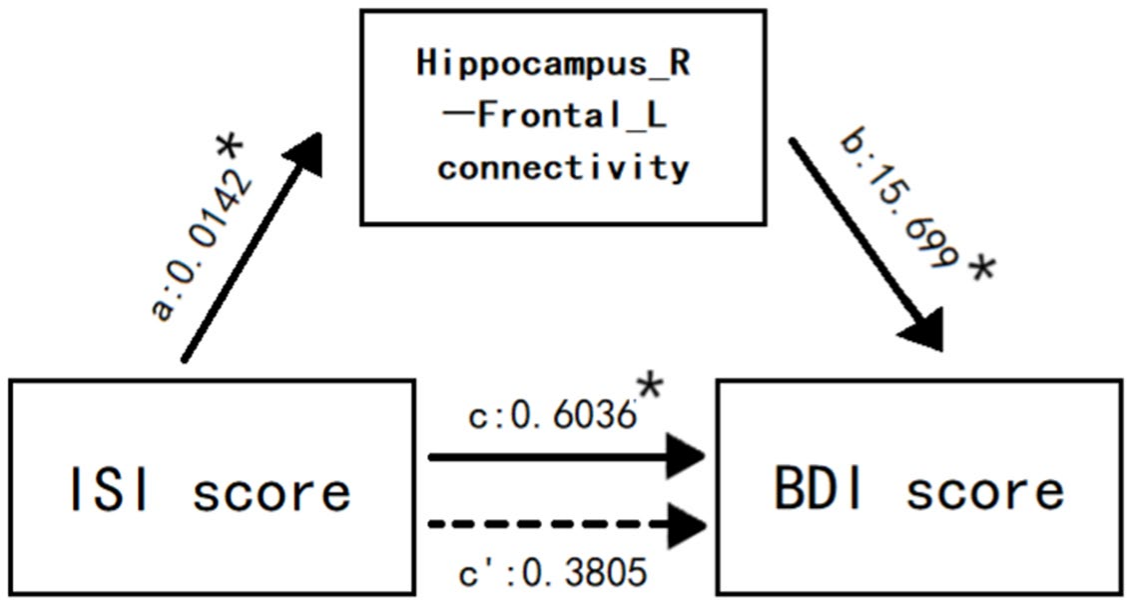
The connectivity of right hippocampus-left inferior frontal gyrus mediates the relationship between ISI score and BDI score. Values are unstandardized regression coefficients reflecting the direct (paths a, b, c’) and total (path c) effects of each relationship in the mediation model. * *p* < 0.05.

The connectivity of right hippocampus-left inferior frontal gyrus mediated the relationship between ISI score and BDI score (Figure 5). Mediation Analysis revealed a significant direct effect of ISI score on the connectivity (*B* = 0.0142, *p* = 0.0367), a significant direct effect of the connectivity on BDI score (*B* = 15.699, *p* = 0.0151), and a significant total effect of ISI score on BDI score (*B* = 0.6036, *t* = 0.0333). However, when the connectivity was taken into account as a mediating variable, the direct effect of ISI score on BDI score was no longer significant (*B* = 0.3805, *p* = 0.1689), indicating the existence of a specific mediated effect. Additionally, the bias-corrected bootstrapping test for the indirect effects of ISI score on BDI score was significant using connectivity as a mediator (*B* = 0.2231), and the 95% confidence intervals (0.0177,0.5077) indicated a specific mediated effect. In other words, the connectivity of right hippocampus-left inferior frontal gyrus accounted for a significant portion of the relationship between insomnia and depression.

## 4. Discussion

Most patients with CID at the sleep medicine clinic who consult a doctor have been noted with a depressive mood. After experiencing long-term insomnia, most patients suffer from MDD, as shown in the flowchart (Figure 1A), which was also confirmed following our subsequent survey (Figure 1B). Therefore, this study selects patients with CID comorbid MDD. We found the reduced CBF and functional alterations in cerebellar and hippocampal regions of patients. That may be involved in the comorbidity of CID and MDD.

Most previous studies of CBF in insomnia have used the sleep deprivation experiment. Participants presented with reduced CBF in the hippocampus, fusiform gyrus, prefrontal cortex. However, the study focusing on CBF in patients with CID are currently scarce. So we know little about CBF in patients with CID. Studies of fMRI have found abnormalities in default networks, negative emotional networks, and reward networks in insomnia. Some brain regions with abnormal FC are closely related to the regulatory functions of sleep and emotion, such as the amygdala, insula, thalamus. Studies of CBF and fMRI in MDD patients were more numerous. They found CBF and FC abnormalities in several brain regions including the frontal lobe, thalamus and anterior cingulate cortex. These brain regions are involved in the mechanisms of emotion regulation. In addition, some studies have explored the relationship between age, gender and the CBF in patients with MDD. The CBF of patients shows different manifestations according to age and gender. We reduced those influences by recruiting participants with balanced age and gender ratios in our study. Furthermore, genes play an important role in the pathogenesis of MDD. It is necessary to explore the mechanism of MDD and CBF changes through the perspective of genes. And the current study has made some new findings (Sun et al., 2023). In our study, the cerebellum was found to present a large area of reduced CBF. And it was associated with insomnia and depression in patients. We also found abnormal FC in hippocampal regions. And it mediates the development of CID to MDD. These findings may be different from previous studies.

Our study’s findings demonstrated a reduction in CBF in the cerebellum of the observed patients, which was located in a large area containing the left cerebellum, right cerebellum, and vermis. The cerebellum’s anterior and posterior lobes also showed reduced CBF. The brain region with reduced CBF was observed to be very large. In this regard, we posited that the reduction of CBF in the cerebellum can serve as a clear biomarker for future studies. Previous studies have found a reduction in CBF in the cerebellum. In a study on shift workers (Park et al., 2019), workers with chronic circadian rhythm disorders developed sleep and emotional disorders. Specifically, their right cerebellar CBF decreased and was negatively correlated with depression scores. In addition, the decrease in cerebellar CBF can also be observed in elderly patients with depression (Liao et al., 2017). Physiologically, the cerebellum mainly plays a role in the regulation of physical movement. However, it also plays a role in regulation of the sleep–wake state and sleep rhythm (Canto et al., 2017). Previous studies have observed changes in sleep rhythm and sleep disorders in patients with cerebellar dysfunction (Song and Zhu, 2022). In an animal experiment, excising the cat cerebellum led to daytime sleepiness (Cunchillos and De Andrés, 1982). That may be related to certain nerve cells and nuclei in the cerebellum, whose nerve impulses may have an impact on sleep. The cerebellum is also closely related to depression. Cerebellar abnormalities have been observed in a plethora of mental disorders (van Dun et al., 2022). In addition, cerebellar lesions may also induce mental disorders such as depression (Frazier et al., 2022). Various studies have suggested that this is due to the close connection between numerous nerve cells or nuclei in the cerebellum and the emotional control brain region (Moreno-Rius, 2019). The cerebellum takes part in emotional regulation by influencing nerve impulses in other brain regions. For example, studies have found that the cerebellum can interfere with the functional activities of the mesencephalon, thereby triggering depression (Baek et al., 2022). Correlation analysis were noted that the decrease in cerebellar CBF was negatively correlated with severity of insomnia and severity of depression. In general, we found that the more severe insomnia and depression, the less cerebellar CBF in patients.

Upon FC analysis, it was found that the connectivity of the left cerebellum-left fusiform gyrus as well as that of the left cerebellum-left occipital lobe were reduced. Meanwhile, connectivity of the left cerebellum- right putamen was noted to be increased. The functions of the fusiform gyrus are mainly related to face recognition, object recognition, and word reading (Weiner and Zilles, 2016; Spagna et al., 2021; Beelen et al., 2022). In prolonged insomnia or depression, patients may presumably report unresponsiveness and diminished face recognition. In previous studies, reduced FC (Gong et al., 2020) and morphological atrophy (Chen et al., 2021) of the fusiform gyrus have been observed. However, the fusiform gyrus morphology has also been shown to be restored when depressive symptoms are alleviated (Xiong et al., 2019). The occipital lobe is generally thought to be involved in visual function, including simple visual functions and visual functions that are both complex and abstract, such as human dreams. Insomnia patients usually have more dreams. The abnormal changes in occipital lobe activity may also be observed. In previous studies, abnormal alterations in insomnia patients with neural activity in the occipital lobe can often be noted, which may be due to disrupted sleep architecture and sleep rhythm. Meanwhile, in patients with depression, abnormal presentation in the occipital lobe can be observed, particularly abnormalities in occipital lobe morphology, where occipital lobe bowing is a distinctive finding (Maller et al., 2014). The putamen is involved in a series of complex functions. Such functions include the formation of behavioral habits, consolidation of memories, recognition of facial expressions, and recognition of the number and shape of objects. Patients with long-term insomnia and depression often experience memory loss, reduced learning ability, and reduced responsiveness. Hence, it is not surprising to observe putamen abnormalities in such patients who may present with both functional activity (Zou et al., 2021; Talati et al., 2022) and morphological abnormalities (Lu et al., 2016) involving the putamen. Our study showed that the connectivity of the left cerebellum- right putamen was demonstrated a positive correlation with both insomnia and depression severity, which was a manifestation of the functional abnormalities in the putamen.

Reduced CBF was also noted in the right hippocampus and left parahippocampal gyrus region. Generally speaking, the hippocampus is primarily associated with human memory, learning, and cognitive function. However, it has been shown to be involved in learning and adaptation in human social activities (FeldmanHall et al., 2021), enabling humans to produce feelings and experiences and form abstract memories (Montagrin et al., 2018). Of course, there is also an accompanying generation of emotions during such processes. The parahippocampal gyrus region is located adjacent to the hippocampus, which together with the hippocampus carries out memory and cognitive functions (Aminoff et al., 2013). Moreover, the parahippocampal gyrus region plays an important role in spatial scene cognition (Baumann and Mattingley, 2016) and word association processing (Diana et al., 2013). The parahippocampal gyrus region, together with other structures, including the hippocampus, mammillary body, anterior thalamic nuclei, and cingulate gyrus, forms the Papez circuit, which is an important neural basis by which humans achieve emotional expression. The hippocampus and parahippocampal gyrus region participate in emotional regulation through the Papez circuit. In addition, the hippocampus and parahippocampal gyrus region are components of the default mode network and salience network, allowing them to play a role in emotion via brain networks. In this study, reduced CBF was noted in the right hippocampus and left parahippocampal gyrus region of the examined patients, which were negatively correlated with HAMD scores. That may be related to abnormal changes in multiple aspects of the hippocampal region. Firstly, both long-term insomnia and depression may cause a reduction in the number of nerve cells and neuroglial cells in the hippocampal region (Chen et al., 2020). Reduced CBF may be a manifestation of damage to neural structures. Secondly, the hippocampal region plays an important role in the regulation of sleep and mood, which is an important part of the neural pathway, such as the papez circuit, default mode network, and salience network. When abnormal reductions in neural activity occur in hippocampal regions, they tend to be accompanied by a reduction in CBF. In addition, abnormal neurotransmitter and hormone levels in the hippocampal region may also contribute to abnormal CBF. Previous studies have found that increased cortisol levels are closely associated with hippocampal atrophy in patients with depression (Lebedeva et al., 2018). And the reduced melatonin levels can lead to impaired neural synapses in the hippocampal region (Li et al., 2022). That may serve as a potential mechanism for reduced CBF.

The connectivity of the right hippocampus-left frontal lobe was found to be increased, which was positively correlated with both the ISI and BDI scores of the patients. By conducting a mediation analysis, the connection was shown to mediate the development of insomnia to depression. Essentially, the connection was found to be strongly associated with insomnia and depression. The region of the brain is roughly in the left inferior frontal gyrus (Brodman 47). Generally, it is mainly responsible for processing grammar. However, recent studies have found that it is also involved in the generation of abstract concepts (Della et al., 2018). Previous studies have found abnormal FC in the left inferior frontal gyrus in patients with depression and other mental disorders (Quide et al., 2018; Zhang et al., 2022). Impaired nerve fibers (Kang et al., 2018) and abnormal FC (Zhao et al., 2019) in the left inferior frontal gyrus has also been observed in insomnia patients. The cause of this abnormal FC in the left inferior frontal gyrus may be related to the central executive network, which is known to be involved in the regulation of sleep and mood. The frontal lobe is an important component of the central executive network. The connectivity abnormalities in the left inferior frontal gyrus exemplify abnormalities in certain sleep and emotion regulatory mechanisms represented by the central executive network (Tozzi et al., 2020; Wei et al., 2020). Since the frontal lobe is involved in both the regulation of sleep and emotion, when abnormalities occur in the frontal lobe, insomnia and depression may arise simultaneously.

In addition, the connectivity of the right hippocampus-right paracentral lobule as well as that of the right hippocampus-right precentral gyrus were found to be decreased. In particular, the connection of the right hippocampus-right paracentral lobule was shown to be negatively correlated with both PSQI and HAMD scores. It is generally accepted that the function of the paracentral lobule is related to lower limb movement. However, the abnormal FC of the paracentral lobule have been reported in several studies in patients with insomnia (Bai et al., 2022) and depression (Zhang et al., 2021). This implies that long-term insomnia and depression can damage the paracentral lobule. The paracentral lobule may also be involved in the regulation of mood and sleep. Furthermore, the precentral gyrus is closely related to somatomotor function. When dysfunctional, it indicates that patients may have developed somatic symptoms. Somatic symptoms of depression are relatively common in certain patients with severe conditions. Accordingly, abnormal alterations in the precentral gyrus due to insomnia (Lanza et al., 2015) and depression (Liu et al., 2021) may explain some of the somatic symptoms in such patients.

There are several limitations in our research. Firstly, the limited number of participants may affect the credibility of experimental results as CBF and FC are susceptible to various factors. That may be difficult to avoid the influence of some confounding factors due to the small number of participants. Secondly, only once magnetic resonance imaging scanning was performed on the participants in our research, making it impossible to see how insomnia and depression gradually affect the brain. Thirdly, combining patient’s hormone levels, brain electrophysiological activities, genetic data, gene expression with CBF and disease also helps with the research. Exploring their relationship with the occurrence of diseases may be our next research topic. Finally, the results obtained through our study are expected to provide assistance to future researchers.

## Data availability statement

The raw data supporting the conclusions of this article will be made available by the authors, without undue reservation.

## Ethics statement

The studies involving human participants were reviewed and approved by Research Ethics Committee of the 960th Hospital of the People's Liberation Army Joint Logistic Support Force. The patients/participants provided their written informed consent to participate in this study. Written informed consent was obtained from the individual(s) for the publication of any potentially identifiable images or data included in this article.

## Author contributions

KL and KX contributed to conception and design of the study. MX organized the database. BL and SQ performed the statistical analysis. YN, QW, SW, YW, CC, ZL, and YJ wrote sections of the manuscript. All authors contributed to the article and approved the submitted version.

## Funding

This study was supported by the Basic Research Key Program, Defence Advanced Research Projects of PLA (2019-JCQ-ZNM-02), the Clinical Medical Science and Technology Innovation Program of Jinan City in 2020 year (grant numbers 202019022), the Medical and Health Science and Technology Program of Shandong Province in 2021 year (grant numbers 202109041050).

## Conflict of interest

The authors declare that the research was conducted in the absence of any commercial or financial relationships that could be construed as a potential conflict of interest.

## Publisher’s note

All claims expressed in this article are solely those of the authors and do not necessarily represent those of their affiliated organizations, or those of the publisher, the editors and the reviewers. Any product that may be evaluated in this article, or claim that may be made by its manufacturer, is not guaranteed or endorsed by the publisher.
